# Neurons cytoskeletal architecture remodeling during the replication cycle of mouse coronavirus MHV-JHM: a morphological in vitro study

**DOI:** 10.1186/s12917-023-03813-y

**Published:** 2024-01-09

**Authors:** Michalina Bartak, Piotr Bąska, Marcin Chodkowski, Beata Tymińska, Marcin W. Bańbura, Joanna Cymerys

**Affiliations:** 1 Division of Microbiology, Department of Preclinical Sciences, Institute of Veterinary Medicine, Warsaw University of Life Sciences, Ciszewskiego 8 St., Warsaw, 02-786 Poland; 2 Division of Pharmacology and Toxicology, Department of Preclinical Sciences, Institute of Veterinary Medicine, Warsaw University of Life Sciences—SGGW, Ciszewskiego 8 St., Warsaw, 02-786 Poland; 3 Laboratory of Nanobiology and Biomaterials, Military Institute of Hygiene and Epidemiology, Kozielska 4 St., Warsaw, 01‐063 Poland

**Keywords:** MHV-JHM, Neurons, Microtubules, Actin filaments, Neurotropism

## Abstract

Nowadays, the population is still struggling with a post-COVID19 syndrome known as long COVID, including a broad spectrum of neurological problems. There is an urgent need for a better understanding and exploration of the mechanisms of coronavirus neurotropism. For this purpose, the neurotropic strain of mouse hepatitis virus (MHV-JHM) originating from the beta-coronavirus genus, the same as severe acute respiratory syndrome coronavirus 2 (SARS-CoV-2), has been used. The role of the cytoskeleton during virus replication in neurons in vitro was determined to understand the mechanisms of MHV-JHM neuroinfection. We have described for the first time the changes of actin filaments during MHV-JHM infection. We also observed productive replication of MHV-JHM in neurons during 168 h p.i. and syncytial cytopathic effect. We discovered that the MHV-JHM strain modulated neuronal cytoskeleton during infection, which were manifested by: (i) condensation of actin filaments in the cortical layer of the cytoplasm, (ii) formation of microtubule cisternae structures containing viral antigen targeting viral replication site (iii) formation of tunneling nanotubes used by MHV-JHM for intercellular transport. Additionally, we demonstrated that the use of cytoskeletal inhibitors have reduced virus replication in neurons, especially noscapine and nocodazole, the microtubule shortening factors.

## Introduction

One of the most tricky and unpredictable family of viruses, *Coronavirideae*, again has forced scientists to investigate crucial stages of viral replication [1]. During the replication cycle, the cytoskeleton plays an essential role and takes part in infection. In healthy, uninfected cells, these dynamic arrays and their associated motor proteins are responsible for the processes of proliferation, migration, differentiation, apoptosis, and intake and transport of cargo by the cell [2]. The cell’s scaffold comprises actin filaments (AFs), microtubules (MTs), and intermediate filaments (IFs). In the context of pathogen invasion, the most crucial role is played by the structures, MTs, as they are involved in the retrograde transport of viral particles, especially in the perinuclear zone, and AFs involved in the movement in the sub-membrane region, cell to cell transport, the entry and egress of virions [3]. Actin is the most abundant protein in eukaryotes. It forms a monomeric and spherical form called globular actin (G-actin), not found outside of myosin, and polymerized in the form of filaments (F-actin) [4]. Actin filaments are prominently accumulated in the sub-membranous region of the cell at its periphery (cell cortex AF). They are capable of forming shorter protrusions such as lamellipodia, membrane ruffle, bladders, and long ones such as filopodia, microvilli, or podosomes (looser bundles) and organized in parallel stress fibers (tightly packed bundles) [4, 5]. Actin filaments also form tubular F-actin-rich structures called TNTs (tunneling nanotubes). They connect the cytoplasm of neighboring and/or distant cells by mediating efficient intercellular communication. They are a kind of long cytoplasmic bridges with the ability to maintain homeostasis in the physiological processes of the cell. Unfortunately, viral particles and other pathogens use these formations at early or late stages of the life cycle, promoting viral spread. The usage of TNTs has been documented for a diverse group of viruses such as retroviruses, vaccinia virus, influenza A virus, human metapneumovirus, human herpes virus type I, and severe acute respiratory syndrome coronavirus 2 to promote viral entry, virus trafficking, and cell-to-cell spread. Especially neuron-neuron transfer of pathological Tau protein assemblies and prion-like proteins in neurodegenerative diseases [6–9].

Microtubules are ultrathin structures, unbranched cylinders made of alpha and beta-tubulin heterodimers that form the wall of protofilaments. In all eukaryotes, they are responsible for essential functions in the cell: segregation of chromosomes into daughter cells at cell division as a significant component of the mitotic spindle, for transport of vesicles and organelles within the cytoplasm, the highly asymmetric morphology of neurons and, together with MTs and IFs, for maintaining the dynamic spatial organization of the cytoplasm in the cell [4, 10–12]. Microtubules are distributed in the cytoplasm in the form of evenly spaced clusters. They form the axons and dendrites of neurocytes, the cytoplasmic protrusions of cells, e.g., fibroblasts, and the pseudopodia in protozoa. Nerve cells are particularly rich in microtubule bundles of the interphase cytoplasm. The structure of the cytoskeleton in neurons is extremely dynamic and is involved in axon transport and proliferation. Microtubules originate at sites called microtubule organization centers (MTOCs), including centrosomes. These structures are where the beginning of microtubule construction occurs. These centers are common organelles present in the perinuclear region, as well as Golgi Apart (GA). MTOCs are suspected of determining the proper location of GAs [4, 13]. Significant is the fact of interaction of microtubules with actin filaments which is possible due to the presence of MAP (microtubule associated proteins) molecules. The level of its phosphorylation influences the formation of branched structures of the cytoskeleton and 3D networks [14]. Moreover, the role of these connections and the MAP molecule is significant in viral infection. Many viruses have encoded their own MAPs to manipulate MT networks directly. These are human immunodeficiency virus type 1, Kaposi’s sarcoma-associated herpesvirus, Epstein-Barr virus, African swine fever virus, human herpes virus type 1, murine norovirus, murine coronavirus [15–23].

As early as 1970, it was discovered by electron microscopy that viral particles localize within cytoskeletal elements, and the effect on cross-linking architecture negatively affects viral replication [24]. Why is it important to know about the changes occurring in the cytoskeleton of cells? Especially in the context of cells of the nervous system, the dysfunction that occurs, the distortion of the architecture of the cytoskeleton leads to severe diseases, including neurodegenerative diseases such as Alzheimer, Parkinson, amyotrophic lateral sclerosis, tauopathies, Huntington’s disease, and Charcot Marie tooth disease [25–27]. The situation is more serious when changes in the cytoskeleton are induced by neurotropic viruses such as some coronaviruses, including the titular MHV-JHM and another well-known beta-coronavirus representative, SARS-CoV-2. Since 2020 cases of common neurological symptoms have been reported, including loss of smell and taste. Less common symptoms were seizures, stroke, and isolated cases of Guillain-Barre syndrome (GBS, acute demyelinating inflammation with coexisting axonal motor neuropathy) [28–30]. According to a recent study, long-term effects caused by SARS-CoV-2 infection include a significant decrease in the brain’s gray matter, changes in markers of damage to tissue connected to the primary olfactory cortex, and a significant reduction in overall brain volume in affected patients [31].

Diagnosed, with increasing frequency, long-term neurological complications due to SARS-CoV-2 infection oblige scientists to intensify research on the neurotropism and neuropathogenicity of coronaviruses. For this purpose, it seems necessary to create new in vitro models suitable for studying the molecular mechanisms of coronavirus neuropathogenicity. One of the coronaviruses that has already contributed to neurodegeneration research is mouse hepatitis virus, specifically the JHM strain (MHV-JHM) [32–34]. A broad spectrum of tissue tropism characterizes MHV strains, and single isolates cause respiratory, gastrointestinal, or CNS diseases [35]. The pulmonary (polytropic) strains of MHV: MHV-1, MHV-2, MHV-3, MHV-JHM (MHV-4), MHV-A59, and MHV-S, replicate initially in the respiratory and olfactory epithelium of the nasal cavity, then develop viremia and spread to the lungs, liver, bone marrow, brain, lymphoid tissue, and reproductive organs. Enterotropic strains, such as MHV-D, MHV-DVIM, MHV-Y, and MHV-RI, mainly infect the gut and can spread to the liver, lymphoid tissue, and spleen [36–38]. Neurotropic strains are the most studied due to their ability to cause acute encephalomyelitis with or without chronic axonal demyelination [34, 37, 39].

**Fig. 1:**
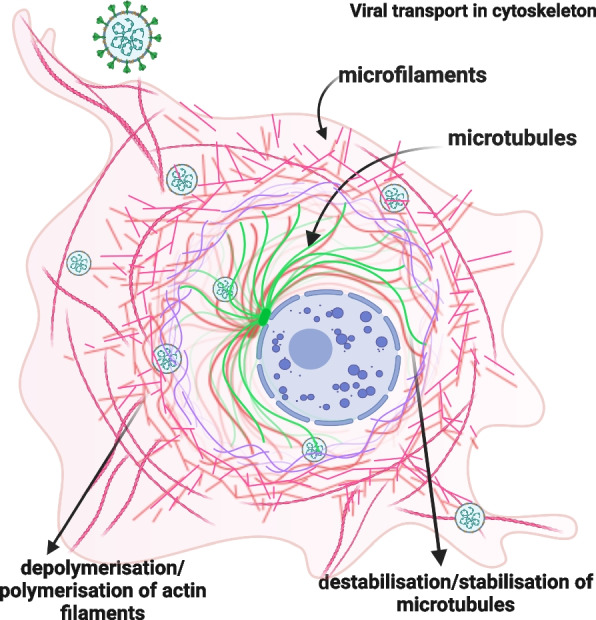
Schematic presentation of MHV-JHM entry and transport. Possible events occurring during virion transport in the cytoskeleton. Own work created with Biorender

Considering various virus-cytoskeleton relationships presented above and the still unknown facts about the interaction of coronaviruses with cytoskeletal structures (especially in nerve cells), we have attempted to characterize the morphological characteristics of microfilaments and microtubules and to check the effect of selected chemicals affecting the disruption of cytoskeletal distribution on the efficiency of MHV-JHM infection in primary neuron cells derived from Balb/c(H-d^2^) mice (Fig. 1).

## Methods

### Primary neuronal cell culture

Balb/c(H-2^d^) mice were used to establish the primary culture of murine neurons using the method by Cymerys et al. [40]. Balb/c mice aged 6–8 weeks were purchased from Animal House of Mossakowski Medical Research Institute Polish Academy of Sciences (Warsaw) and were handled in accordance with the guidelines regarding the use and care of laboratory animals. All actions involving live animals were performed according to Polish Local Ethics Committee guidelines and conformed to applicable international standards. Pregnant female mice (16–19 days post mating) were sacrificed in general anesthesia induced with 2%-3% isoflurane in an induction chamber (MiniVac Complete Anesthesia Systems, Harvard Apparatus). After sedation mice were sacrificed by cervical dislocation. Fetuses were removed and decapitated for brain collection. Then isolated cerebral hemispheres from fetal’ brains were washed three times in cold HBSS solution (10× Hanks Buffer; Life Technologies Waltham MA, USA) and treated with 2,5% EDTA-free trypsin solution at 37°C in 5% CO_2_ for 15 min. Again, cells were washed three times in a warm HBSS solution after incubation and mechanically homogenized using a pipette. After suspending and counting, cells were plated onto poly-L-lysine or poly-D-lysine with laminin-coated coverslips for immunofluorescent staining and without for RT-qPCR replication analysis at a density of 5 × 10^4^ neurons per well (3.6 cm^2^). Primary murine neurons were cultured in B-27 Neuron Plating Medium, consisting of the neurobasal medium, B-27 supplement, 200 mmol/l of glutamine, 10 mmol/l of glutamate, and penicillin/streptomycin antibiotics (1%) with 5% supplement of fetal bovine (5%) and 5% equine serum (5%) (Gibco Life Technologies, Waltham MA, USA). To avoid propagation of non-neural cells, cultures were maintained in a growth medium supplemented with 10 μM cytosine β-D-arabinofuranoside (after 3 days for 24 h) (Sigma-Aldrich, Darmstadt, Germany). Subsequently, the medium was removed and replaced with Neuron Feeding Medium (B-27 Neuron Plating Medium without glutamate; Life Technologies Waltham MA, USA). In such conditions, neurons were maintained for the next 8 days prior to analysis, infection, and treatments with inhibitors at 37°C with 5% CO_2_.

### MHV-JHM maintenance

Mouse coronavirus (MHV, mouse hepatitis virus), neuropathological strain MHV-JHM [ATCC-VR-76513] was propagated and in mouse hepatocyte cell line [NCTC, ATCC-CCL-9.1]. The median tissue culture infectious dose (TCID_50_) was calculated using the Spearman-Kärber method [41]. Aliquots were stored at -70°C. All studies were conducted with the virus stock at the second passage level and titer of 10^7.8^ TCID_50_/mL.

### Primary cell culture infection

Primary neuronal cell cultures were infected with MHV-JHM strain (MOI = 1.25) for 60 min at 37°C. After incubation, the inoculum was removed, washed with PBS, and a fresh culture medium was added. Subsequently, infected cells were incubated for 2, 24, 48, 72, and 168 h at 37°C with 5% CO_2_.

### Inhibitors and cell viability assay (XTT)

Mouse hepatocytes (ATCC-CCL-9.1) were cultured in a 96-well plate at 10,000 cells/well and incubated until fully confluent (24 h) at 37°C and a 5% CO_2_-enriched atmosphere. After full confluence was achieved, the culture fluid was aspirated, and a suspension of each serine protease and cytoskeletal inhibitors (Table 1) were added at a volume of 100 µl per well. The cultures were incubated for 24 h at 37°C and in an atmosphere enriched with 5% CO_2_. After incubation, 50 µl of XTT labeling mixture (Roche, The Cell Proliferation Kit II (XTT), Darmstadt, Germany) was added to each well. After a 4-h incubation (37°C, 5% CO_2_), a reading was taken at λ = 450 nm, subtracting the background measured at 600 nm. Individual assays were performed in 3 replicates, and the average absorbance value for each dilution was calculated, followed by the calculation of the percentage of viable cells compared to the positive control (assuming 100% viable cells for the absorbance value of the positive control).

**Table 1:** Inhibitors of cell cytoskeleton used in the study

**Substance**	**Form**	**Therapeutic effect/treatment**
**Nocodazole**	benzimidazole substituted at position 2 by a (methoxycarbonyl)amino group and at position 5 by a 2-thienoyl group	antineoplastic agent, a tubulin modulator, an antimitotic, and a microtubule-destabilizing agent and an inhibitor of various cancer-related kinases.
**Noscapine**	phthalide isoquinoline alkaloid from *Papaver somniferum*	analgesic, antitussive, disruption of the dynamics of microtubule assembly, the inhibition of mitosis and tumor cell death, noscapine does not affect microtubule polymerization, antiviral activity by inhibition of Mpro protease.
**Paclitaxel**	a tetracyclic diterpenoid from the bark of the *Taxus brevifolia*	a mitotic inhibitor used in cancer chemotherapy, an antineoplastic agent, a microtubule-stabilizing agent, and an antineoplastic agent.
**Latrunculin A**	16-membered bicyclic lactone attached to the rare 2-thiazolidinone moiety from *Latrunculia magnifica*	impacts actin polymerization, microfilament organization, and microfilament-mediated processes.
**Cytochalasin D**	cell-permeable fungal toxin; from *Zygosporium mansonii*	potent inhibitor of actin polymerization; disrupts actin microfilaments; activates the p53-dependent pathways; inhibits smooth muscle contraction.

### Immunofluorescence staining and imaging

The immunofluorescence method was used to visualize cell structures and viral antigen. After incubation in desired infection time, primary neuronal cell cultures were washed twice in PBS (Sigma-Aldrich, Darmstadt, Germany), then fixed in 4% PFA (paraformaldehyde, ThermoFisher, Waltham MA, USA) for 30 min. After fixation, the cells were washed twice with PBS solution and further incubated with 0.5% Tween/PBS solution for 10 min at room temperature. Following, the cells were washed twice with PBS solution. The F-actin filaments were stained with 50 μL of TRITC-labelled phalloidin conjugate (500 µg/mL; Sigma-Aldrich) and incubated for 60 min in a wet chamber. Likewise, microtubules were stained for 60 min in a wet chamber with indirect immunofluorescence with Anti-β-Tubulin III antibody produced in rabbit (dilution 1:200, Sigma Aldrich, Darmstadt, Germany) and then visualized with a secondary antibody, Texas Red goat anti-rabbit IgG (dilution 1:2000, Sigma- Aldrich, Darmstadt, Germany) for 60 min. The presence of viral antigen was determined by indirect immunofluorescence, using SARS-CoV/SARS-CoV-2 Spike Protein S2 Monoclonal Antibody (1A9) (ThermoFisher, Waltham MA, USA, dilution 1:250) incubated overnight at 4°C. To visualize the viral antigen, Alexa Fluor 488 anti-mouse IgG was used for 60 min, RT. Additionally, cell nuclei were stained with Hoechst 33258 (ThermoFisher, Waltham MA, USA) for 2 min, RT. Afterward, coverslips were mounted on microscope slides using ProLong Gold Antifade Mounting Medium (ThermoFisher, Waltham MA, USA). Images were acquired in a confocal microscope (Fluoview FV10i, Olympus, Warsaw, Poland), saved in 24-bit.tiff format, and analyzed using FV10i software (Olympus), ImageJ2 (NIH Image, version 1.53q, Bethesda, MD, USA), and Adobe Photoshop CS6 software (Adobe Systems Incorporated, ver. 23.4.1, San Jose, CA, USA).

### Cell treatment method with cytoskeleton-interfering inhibitor drugs

Primary neuron cells after maturation (day 14) and at proper confluence level (min 70%) were pretreated or post-treated with nocodazole 30μM/mL; cytochalasin D 10μM/mL; latrunculin A 10μM/mL; noscapine 75μM/mL; Taxol 10μM/mL, all dissolved in DMSO 1%. During the pre-treatment method, cells were incubated for 1 h with the desired drug at 37°C, 5% CO_2_ cells, then infected with MHV-JHM. After infection, the medium was replaced with a fresh culture medium. In the post-treatment method, cells were infected with MHV-JHM for 1 h at 37°C, 5% Co_2_. Then the cells were washed and left for incubation in a fresh medium containing the listed drugs. Primary neuronal cell cultures were further incubated for 2, 24, 48, 72, and 168 h. Cellular and supernatant fractions from appropriate periods were collected in RLT buffer (Qiagen, Germantown, MD, USA), stored at -20°C, and later used for RNA isolation and reverse transcription quantitative real-time PCR (RT-qPCR) analysis.

### RNA isolation and reverse transcription quantitative real-time PCR (RT-qPCR)

#### Primer design and construction of standard for RT-qPCR

RNA from infected and control cells was isolated using RNeasy Mini Kit (Qiagen, Germantown, MD, USA) followed by cDNA 1st synthesis using random hexamer primer (RevertAid First Strand cDNA Synthesis Kit, ThermoFisher). Product spanning a particular region of the MHV genome (Gen Bank No. AC_000192.1) was amplified using MHV_L_full and MHV_R_full primers (Table 2), followed by electrophoresis and gel extraction. The extracted DNA fragment was used for reamplification and cloned in E. coli using pGEM®-T Easy Vector System (Promega), and recombinant plasmid pGEM-T/MHV_1 was achieved. RT-qPCR (in the total volume of 12 µl) with primers MHV_L_v2 (0.9 µM), MHV_R_v2 (0.9 µM), and probe (0.2 µM) (Table 2) was performed on cDNA from both infected and control cells, as well as on plasmid pGEM-T/MHV_1 construct using TaqMan™ Gene Expression Master Mix (ThermoFisher, Waltham MA, USA). The PCR was performed as follows 50°C – 2 min, 95°C- 10 min, 45 × (95°C – 15 s, 60°C – 1 min).

**Table 2:** Description of primers used during RT-qPCR

**Primer**	**Sequence**	**AC_000192.1 covering region**	**Product length**
*MHV_L_full*	TTGGCTTGTGAGTGACGCCTG	28 571 – 28 591	806 bp
*MHV_R_full*	GCGCATACACGCAATTGAACAT	39 376 - 29 355	
*MHV_L_v2*	GTATGGTATGTGGGGCAGATTA	28 906 – 28 927	88 bp
*MHV_R_v2*	GTTTAATAGACGCAAGGAAGGC	28 993- 28 972	
*Probe 5` 6-FAM 3` TAMRA*	AGTCGCAGTGTGTTTGATGGTCACC	28 935 – 28 959	Not applicable

#### Measurement of MHV copies in samples using RT-qPCR

RNA was isolated from cells using RNeasy Mini Kit (Qiagen, Germantown, MD, USA), followed by RNA concentration measurement using Synergy H1 Microplate Reader (BioTeK) and cDNA 1st synthesis using random hexamer primers (RevertAid First Strand cDNA Synthesis Kit, ThermoFisher, Waltham MA, USA). The cDNA was diluted 40 × in water. In parallel control buffer (CON_buf) was prepared by mixing all necessary rea-gents for RT reaction except from RNA, dNTPs, and random hexamer primer: 14 µl of water, 4 µl of reaction buffer (5 × concentrated), 1 µl of RNA inhibitor and 1 µl of Reverse Transcriptase (RevertAid First Strand cDNA Synthesis Kit, ThermoFisher, Waltham MA, USA) were mixed and incubated 25°C – 5 min, 42°C - 60 min and 70°C – 5 min. The mixture was diluted 40 x, and CON_buf was achieved. The RT-qPCR was performed in 12 µl prepared as follows: 6 µl of 2 × TaqMan™ Gene Expression Master Mix (ThermoFisher, Waltham MA, USA), 2.16 µl of primers mix MHV_L_v2 and MHV_R_v2 (5 µM each), 1.2 µl of probe (2 µM) and 0.64 µl of water were mixed resulting in mixture volume of 10 µl. The mixture was added to the plate wells, and 2 µl of 40 × cDNA was added as matrices. To achieve a standard curve, pGEM-T/MHV_1 was diluted in CON_buf to achieve 1E8, 1E7, 1E6, 1E5, 1E4, 1E3, 1E2 and 33 copies per 2 µl and used as matrices for RT-qPCR. The PCR was performed as follows: 50°C – 2 min, 95°C - 10 min, 45 × (95°C – 15 s, 60° C – 1 min). Additionally, each plate contained a sample with cDNA from infected and control cells serving as positive and negative controls, respectively. Each reaction was performed in triplicate in MicroAmp™ Optical 96-Well Reaction Plate with Barcode using a thermocycler.

### Real-time cell growth analise JuLi^TM^Br

To determine the cellular growth and morphology of primary neurons infected with MHV-JHM, the JuLI^TM^Br Live Cell—system for bright-field analysis (NanoEnTek, Seoul, Korea 2015) was used. When cultured neurons reached about 80% confluency, cells were infected with MHV-JHM as previously described. Images were captured for 168 h with 30 min intervals. The results were obtained and analyzed using JuLI^TM^Br PC software. Uninfected cells were used as a negative control. All images were captured at a × 40 magnification.

### Statistical analysis

The results were statistically evaluated by one-way or two-way analysis of variation (ANOVA) using the Tukey multiple comparisons test or multiple unpaired t test using threshold p value with the Šídák-Bonerroni multiple comparisons correction method. All experiments were done at least in triplicate. These analyses were performed using GraphPad Prism™ version 9.4.0 (453) for macOS software (GraphPad Software Inc., San Diego, CA, USA). Statistical differences were interpreted as significant at *p* ≤ 0.05, highly significant at *p* ≤ 0.01, extremely significant at *p* ≤ 0.001, and insignificant at *p* > 0.05.

To analyze the colocalization of the fluorescence signal derived from TNTs and the viral antigen during the study, a minimum 100 confocal images were used. Images were analyzed using the Fiji BIOP JACoP plug-in. The parameters analyzed were two channels, green fluorescence corresponding to the viral antigen and red fluorescence for the F-actin. The quantitative interpretation of pixel correlation coefficients in the form of threshold parameters of Pearson’s correlation coefficient (PCC) and Manders’ correlation coefficient (M1 and M2) of global statistical analysis were considered for statistical analysis, considering pixel intensity distributions from fluorogram plots [42]. The degrees of correlation were indicated as perfect for values near ± 1; strong for values between ± 0.50 and ± 1; medium for values between ± 0.30 and ± 0.49, and low for values below + 0.29.

## Results

### MHV-JHM productively replicates in neurons

**Fig. 2:**
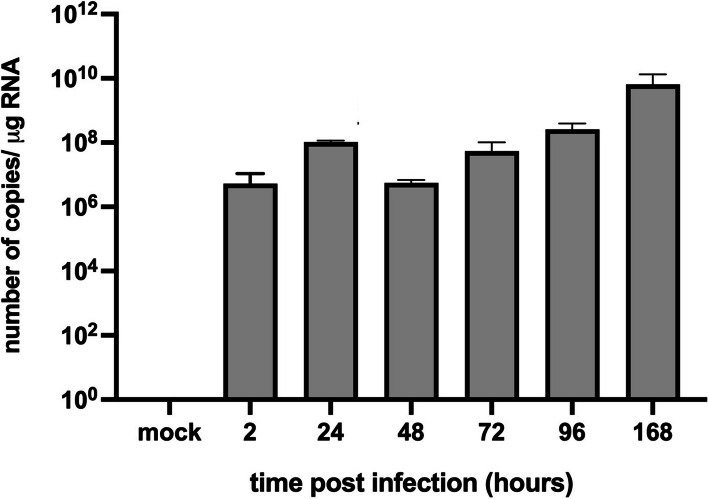
RT-qPCR analysis of MHV-JHM viral RNA copies per µg during 168 h p.i. in murine neurons

**Fig. 3:**
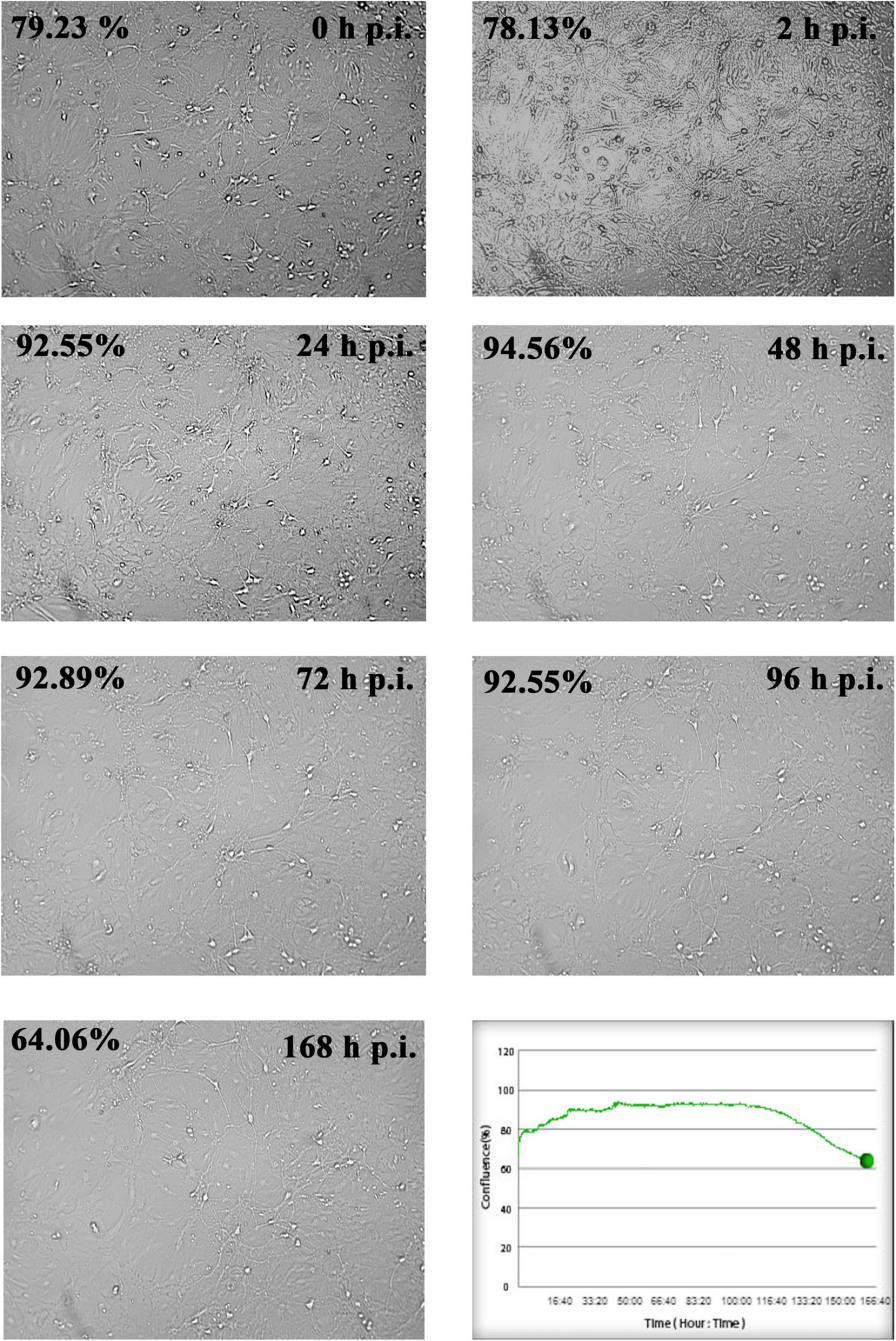
Real-time cell growth analysis of MHV-JHM infected primary murine neurons performed by using live image move analyser JuLi™Br. Cultures were observed from initial seeding for 168 h. The generated graph shows the percentage of cells' confluence level [%] during complete analysis [hours]. All images were recorded every 5 min and analysed monolayer confluence. Objective magnification × 40

The number of copies of viral RNA were determined from the suspension of cells and media from each time post-infection (Fig. 2). In the positive control of neuron cells infected with MHV-JHM, the number of copies per µg RNA has been increasing logarithmically. At the start, after 2 h p.i., the virus copies were at 5.30 × 10^6^ per µg RNA. Then, 24 h p.i. values increased to 1.03 × 10^8^ copies/µg RNA when the complete replication cycle occurred. At 48 h p.i., values decreased by two logarithms (5.60 × 10^6^) and gradually raised, reaching its peak – 6.48 × 10^9^ at 168 h p.i. On the other hand, the morphological analysis done with JuLi™Br live imaging showed that neurons infected with MHV-JHM did not drastically change the confluence of the culture nor the appearance of cells (Fig. 3). During the 168 h of infection, the lower density of cell culture was observed, but the ability to form long protrusions or neuritis were present until the assay’s end. Therefore, there was no visible CPE, and cells did not undergo lysis. The confluence level had been raised until 96 h post infection (92.55%) and started to drop after 120 h p.i., reaching the final confluence of 64.06% at 168 h p.i. (Fig. 3, graph).

### MHV-JHM controls the dynamic processes of the actin cytoskeleton

The effect of MHV-JHM on the cytoskeletal structure of primary cultured mouse neurons manifested in the form of changes in the distribution of filaments.

**Fig. 4:**
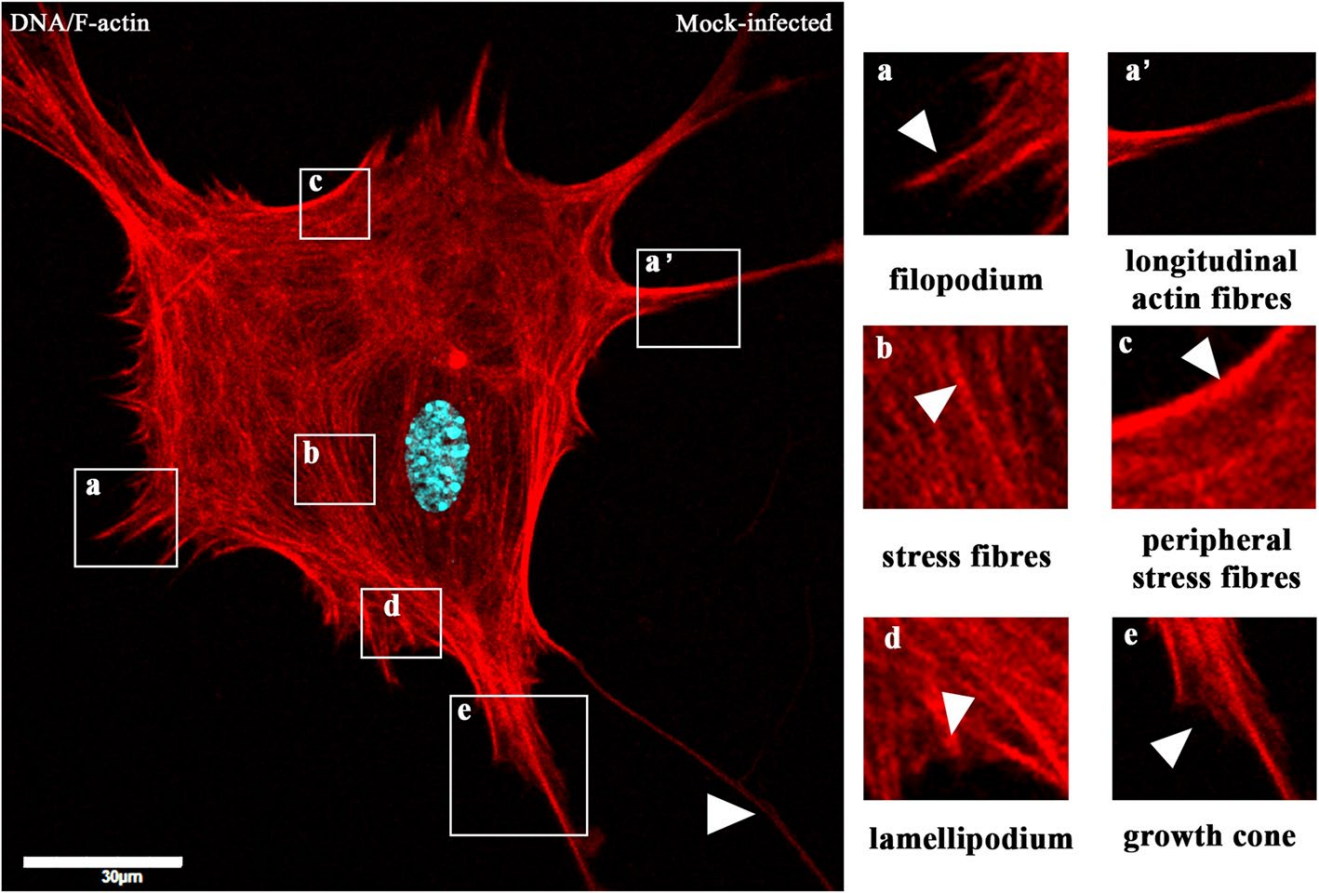
Actin cytoskeleton network morphology of non-infected primary murine neurons. Various forms of actin fibres structures were presented and highlighted by white arrowheads: tunnelling nanotube (main image), filopodium (a) and longitudinal actin fibres (a’), stress fibres (b), peripheral stress fibres (c), lamellipodium (d), and growth cone (e). Indirect and direct immunofluorescence staining; merge images: actin filaments – red; cell nuclei – blue. Microscope magnification 60x, scale 20 μm

**Fig. 5:**
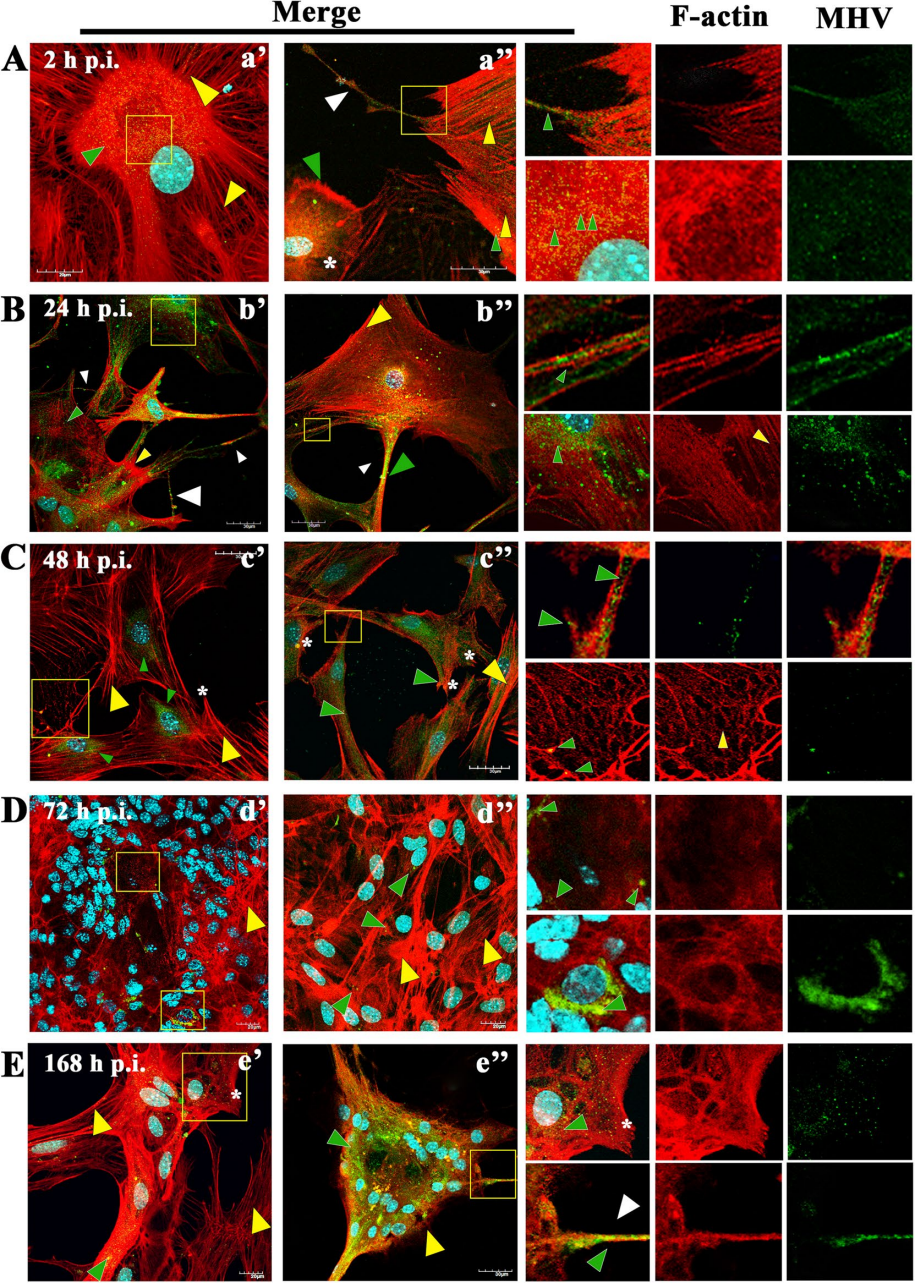
Primary culture of murine neurons infected with MHV-JHM virus. Representative confocal images of neurons obtained at 2 (A,a’,a’’), 24 (B,b’,b’’), 48 (C,c’,c’’), 72 (D,d’,d’’), and 168 h p.i (E,e’,e’’). Green arrowheads point to the presence of viral antigens in actin structures. Yellow arrowheads show areas of changes in f-actin filaments resulting from MHV-JHM infection. White arrowheads point to tunnelling nanotube (TNTs) structures. White asterisk show lamellipodia. Yellow boxes indicate the overlapping presence of actin filaments fluorescence with viral antigen and magnified area. Indirect and direct immunofluorescence staining; merge images: actin filaments - red; cell nuclei - blue; viral antigen - green. Microscope magnification 60x, scale 20 μm

**Fig. 6:**
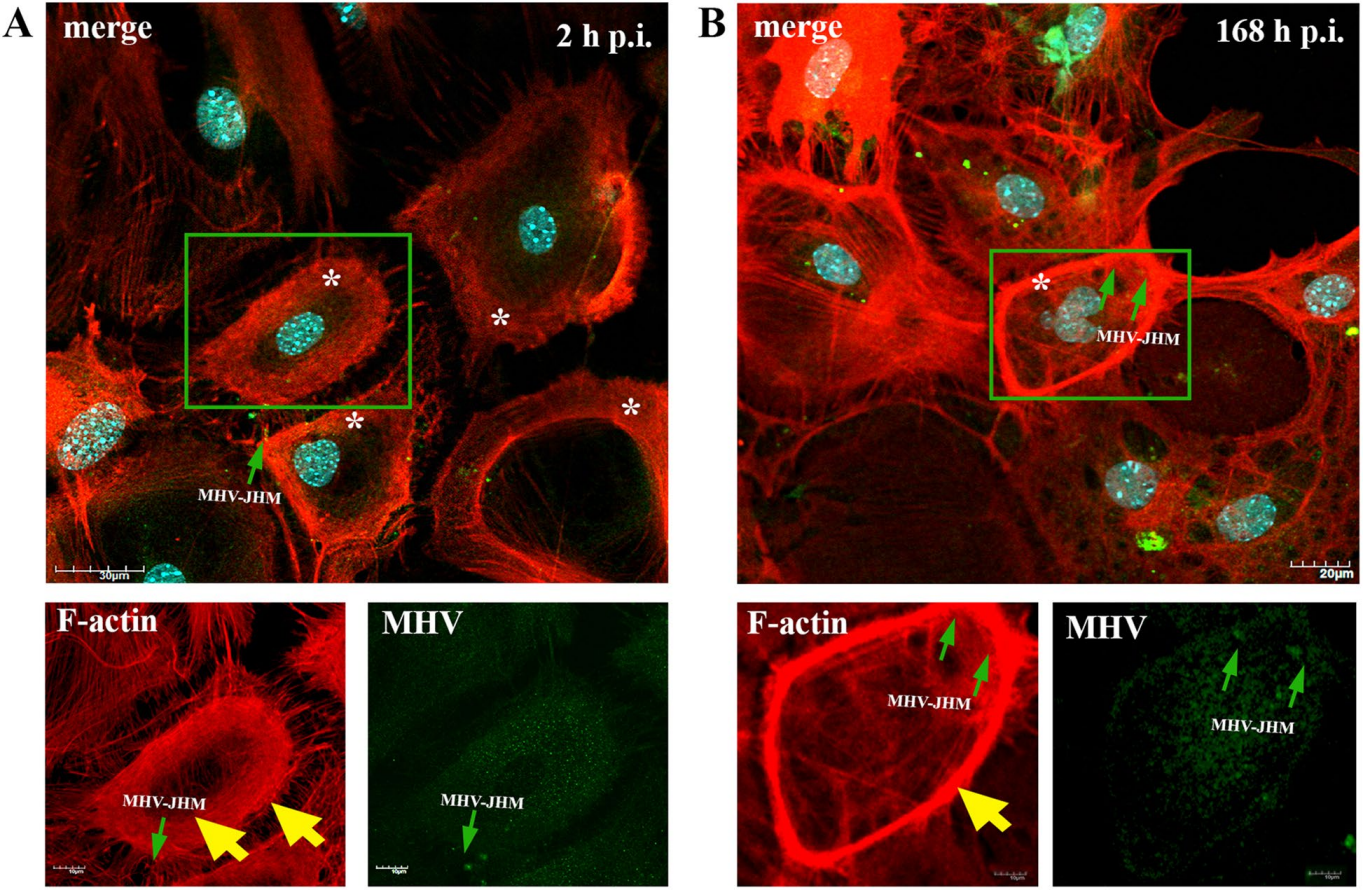
Primary culture of murine neurons infected with MHV-JHM virus. Representative confocal images of neurons obtained at 2 h p.i. (**A**) and 168 h p.i (**B**). White asterisk represents the occurrence of a F-actin ring structures – juxtanuclear ring (**A**) and submembranous ring (**B**); green boxes show magnified areas; green arrows indicate viral antigen; double yellow arrows show juxtanuclear ring and single yellow arrow points submembrane ring. Indirect and direct immunofluorescence staining; merge images: actin filaments - red; cell nuclei - blue; viral antigen - green. Microscope magnification 60x, scale 20 μm and 10 μm

The first changes could be seen as early as 2 h p.i., where, compared to the control (Fig. 4) even arrangement of stress filaments (Fig. 5A, a’,a’’ yellow arrowheads), excessive condensation of actin in the submembranous region (Fig. 5A,a’’ green arrowhead; Fig. 6A, white asterisk) and its thinning in the zone close to the cell nucleus (Fig. 5A,a’’, white asterisk; Fig. 6A, yellow arrow) were noted. Also, the presence of penetrating/moving viral antigens in the long filopodia (Fig. 5A,a’’, white arrowhead) and the accumulation of virion antigen was captured in the perinuclear area, which is the site of target replication in the cytoplasm (Fig. 5A,a’, yellow box, green arrowhead).

The changes have worsened at 24 h p.i. When productive replication occurred, neurons formed many intercellular connections in the form of longitudinal actin filaments - tunnelling nanotubes, to transport viral particles to neighbour cells (Fig. 5B, b’ white arrowheads). It is possible to distinguish the formation of thin TNT bridges and long thicker ones (Fig. 5B, b’,b’’ white arrowheads). In the area marked by the yellow box, the progeny virions were present, moving across the bridge from one cell to another (Fig. 5B, b’’ green arrowheads). Peripheral stress fibres in the form of highly condensed structures were still visible (Fig. 5B, b’, b’’ yellow arrowheads), and a large amount of viral antigen localized in the perinuclear region (Fig. 5B, b’, yellow box, green arrowhead) with a much-diluted structure was still clearly visible.

Interesting morphological changes begin to occur on the second day after infection, where neurons clearly have lost their ability to form long protrusions (Fig. 5C, c’,c’’). Instead, shorter filopodia appeared (Fig. 5C, c’,c’’ white asterisk). Also, the structure of the stress fibres has again become highly polarized fibres in which the viral antigen was present (Fig. 5C, c’,c’’ green arrowheads and yellow arrowheads).

Complete rearrangement and loss of the proper structure of actin filaments and their local defragmentation occurred at 72 h p.i. (Fig. 5D, d’,d’’). The viral antigen was predominantly present in the perinuclear space where the actin structure has been polymerized (Fig. 5D, d’ yellow boxes green arrowheads).

**Fig. 7:**
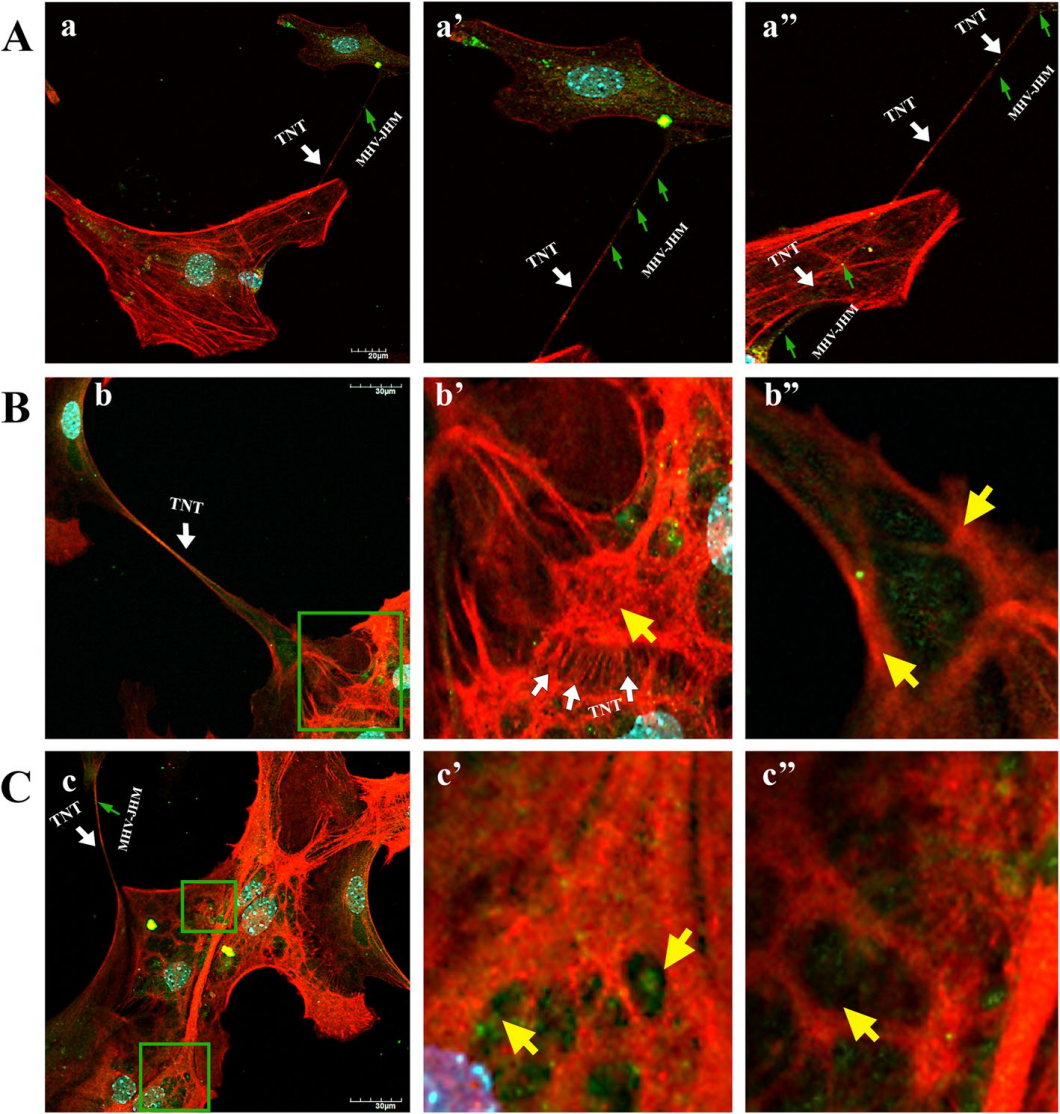
Primary culture of murine neurons infected with MHV-JHM after 168 h p.i.. Representative confocal images of specific filamentous actin structures – tunnelling nanotubes (TNTs) (a,a’,a’’,b,b’,c; white arrows); depolymerised (b’ yellow arrow) and highly polymerised rings structures (b’’ yellow arrows) spider-web-like actin structures (c’,c’’ yellow arrows). Green boxes indicate magnified area. Indirect and direct immunofluorescence staining; merge images: actin filaments - red; cell nuclei - blue; viral antigen - green. Microscope magnification 60x, scale 30 μm and 20 μm

**Fig. 8:**
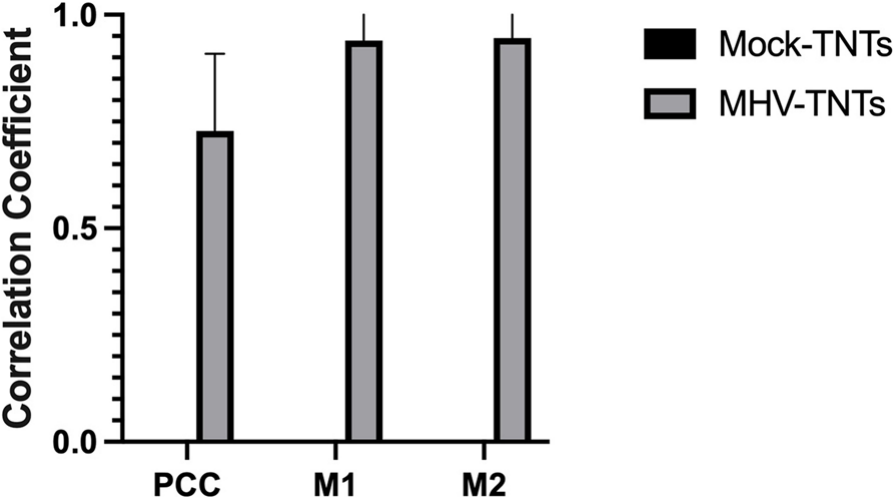
Colocalization analysis of viral antigen present inside tunnelling nanotubule structures. Histograms compared correlations of TNTs with MHV-JHM antigen from the 2 h p.i. until 168 h p.i with uninfected cells (mock-infected) showing Pearson’s correlation coefficient (PCC) and Meander’s coefficients (M1 and M2) from ≥ 100 cells (data represented as mean ± SEM from TNTs structurers). The degrees of correlation were indicated as perfect for values near ± 1; strong for values between ± 0.50 and ± 1; medium for values between ± 0.30 and ± 0.49, and low for values below + 0.29. Obtained with JACoP BIOP analysis

Interestingly, 1 week after infection, the structure of the actin cytoskeleton has restored. (Fig. 5E, e’,e’’). Stress fibres (Fig. 5E, e’ yellow arrowheads), TNTs (Fig. 5E,e’’, yellow box, white arrowhead), and lamellipodia (Fig. 5E, e’ yellow box, white asterisk) could be distinguished. However, further examples of the cytopathic effect in the form of spiderweb-like structure  (Fig. 5E, e’ yellow box) and cell syncytia (Fig. 5E, e’’ yellow arrowhead) appeared. The moving viral antigen have been still present in the actin protrusions (Fig. 5E, e’’ yellow box, green arrowhead). These actin structures were noticeable throughout the infection cycle but were best and most visible 1 week after infection. Such as numerous long actin protrusions and nanotubes in which the moving viral antigen was present (Fig. 7A,B,C; white arrow). A tunneling nanotubule connected two nerve cells transmitting virions between them (Fig. 7A a’’; white arrow). The quantification colocalization analysis of viral antigen and TNTs structure throughout the infection exhibited strong values (PCC = 0.73 ± 0.18; M1 = M2 = 0.93) (Fig. 8). Moreover, interesting spiderweb-like structures appeared with local highly polarized filaments and complete absence or depolarization (Figs. 6B and 7C; green boxes and yellow arrows).

Another interesting phenomenon was also observed. The formation of actin structures, highly polarized filaments forming a circle, was noted during infection at early and late time post infection (Fig. 6A,B). At 2 h p.i., the filaments were polarized in the perinuclear and submembrane area (Fig. 6A, white asterisk; yellow arrows) while at 168 h p.i., a significant condensation of polarized actin filaments were visible, forming a ring-like form in submembrane area (Fig. 6B, white asterisk, yellow arrow). In these structures, high number of viral particles were noted (Fig. 6A,B; green arrows).

### MHV-JHM modulates microtubule structure and uses during transport

**Fig. 9:**
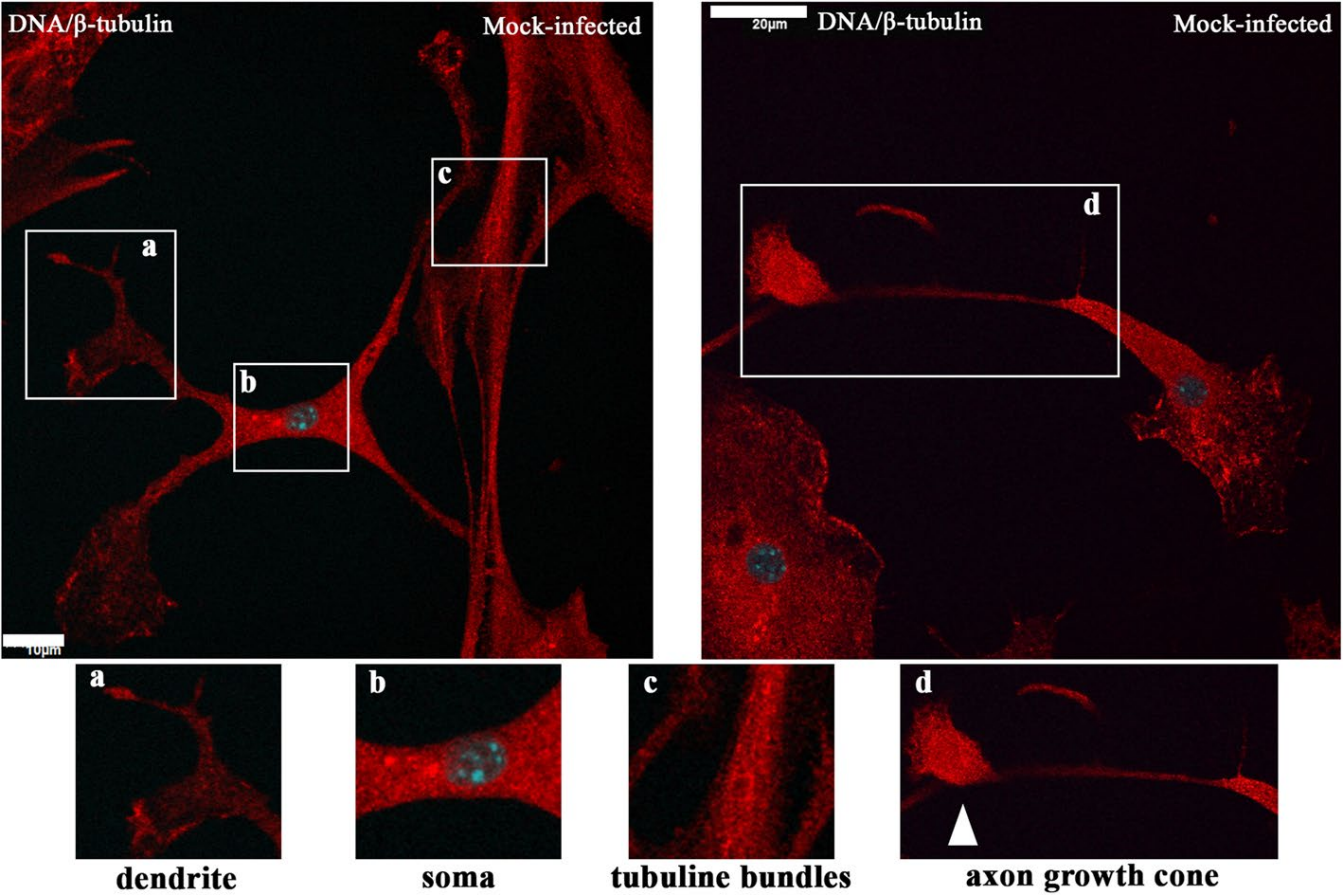
Microtubule network morphology of non-infected primary murine neurons. Various forms of β-tubulin rich structures were presented and highlighted by white boxes: dendrite (a), soma (b), tubulin bundles (c), and axon growth cone (d). Indirect and direct immunofluorescence staining; merge images: β-tubulin- red; cell nuclei – blue. Microscope magnification 60x, scale 10 μm

**Fig. 10:**
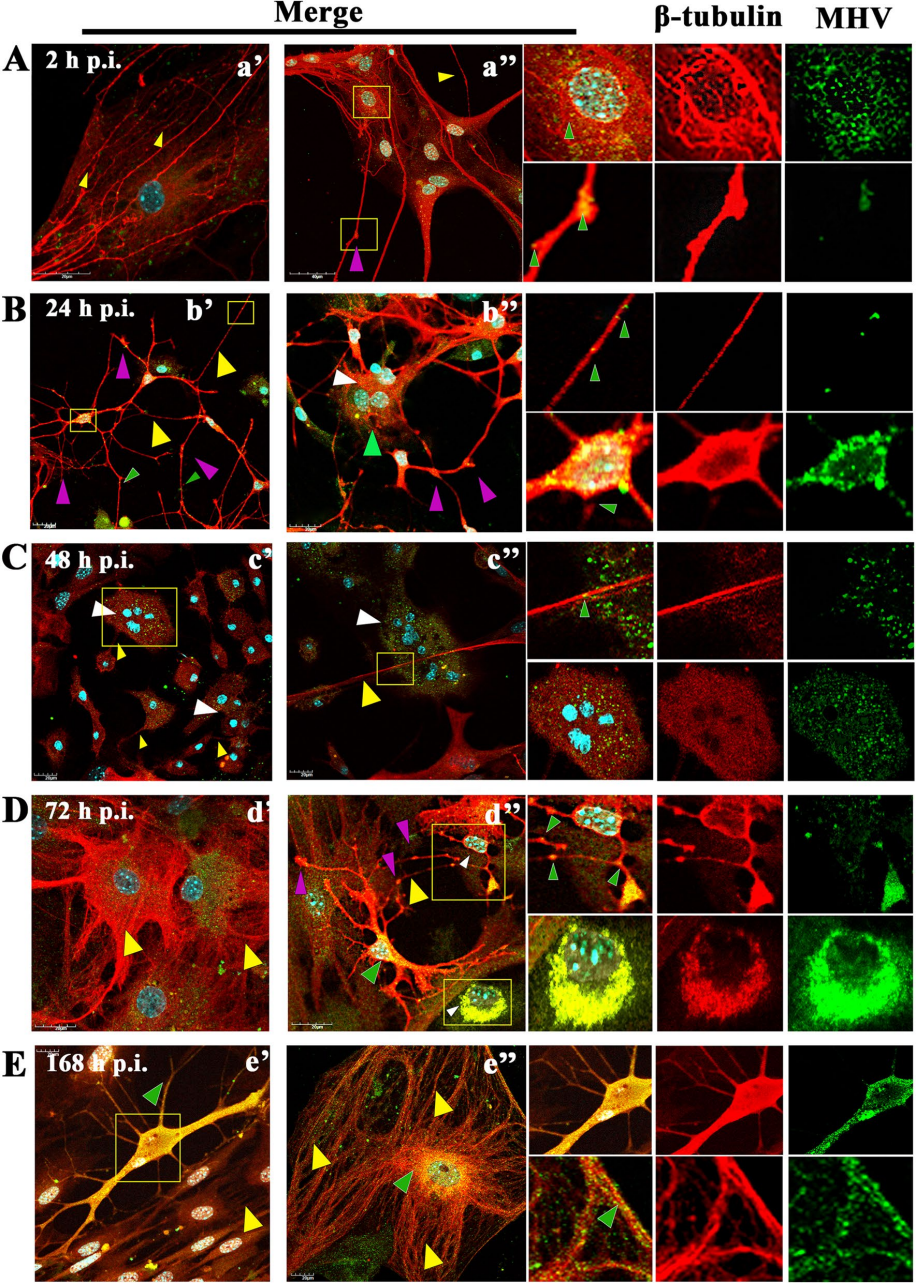
Primary culture of murine neurons infected with MHV-JHM virus. Representative confocal images of neurons obtained at 2 (A,a’,a’’), 24 (B,b’,b’’), 48 (C,c’,c’’), 72 (D,d’,d’’), and 168 (E,e’,e’’) hours post infection. Green arrowheads point the presence of viral antigens in actin structures; Yellow arrowheads show areas of changes in β-tubulin resulting from MHV-JHM infection. White arrowheads point to syncytia formation. Purple arrowheads indicate cisternae microtubule structures. Yellow boxes indicate the overlapping presence of β-tubulin fluorescence with viral antigen. Indirect and direct immunofluorescence staining; merge images: β-tubulin - red; cell nuclei - blue; viral antigen - green. Microscope magnification 60x, scale 20 μm

Microtubules are less involved in virus entry into the cell. In contrast, MTs in neurons facilitate viral transport along the neuron’s cell body and axonal terminals. This can be seen as early as 2 h p.i. as viral particles were moving ‘surfing’ inside MTs (Fig. 10Aa’’). In the box highlighted in yellow, attention was drawn to the accumulation of viral antigen in the perinuclear space and its presence in the neurite (Fig. 10Aa’’, yellow box, and green arrowheads). Also, viral antigen was visible in perinuclear area (Fig. 9Aa’’, yellow box, and green arrowheads).

Progressive accumulation of viral antigen was observed after the first day after infection. On images, it was manifested as yellow fluorescence present mainly in the soma region (Fig. 10Bb’, yellow box, green arrowhead). The dots of green fluorescence present in numerous dendrites likely represented progeny virions moving between neurons (Fig. 10Bb’, yellow box, green arrowheads). Early syncytium formation was also detected after 24 h p.i. (Fig. 10Bb’’, green and white arrowhead).

Compared to control (Fig. 9), microtubule architecture changes occurred after 48 h p.i. Microtubules radiated toward the forming syncytium (Fig. 10Cc’,c’’, yellow square, yellow arrowheads, white arrowhead) were noted. On the first image, a loss of dendrites, axons and a pronounced rounding of the neuron cell were seen (Fig. 10Cc’). In places, as captured in the second photo, a long protrusion is present in which the progeny virion was moving (Fig. 10Cc’’, yellow arrowhead, green arrowheads).

On the other hand, on the third day after infection, a restoration of the neurons’ ability to form protrusions was observed (Fig. 10Dd’,d’’, yellow arrowheads). However, compared to control (Fig. 9), the structure of MTs became depolymerized, facilitating the distribution of viral proteins (Fig. 10Dd’,d’’, yellow arrowheads). The second picture showed strong condensation of microtubules at the site of viral antigen and formation of bulges in dendrites - a possible site of release of progeny virus (Fig. 10D,d’’, yellow square, green arrowheads). Syncytia at 72 h p.i. were still present with a clear ring of viral antigen around fused cell nuclei (Fig. 10D,d’’, yellow squares, white arrowheads).

Interestingly similar to what was described for actin filaments, 1 week after infection, there was no loss of cellular protrusions, microtubule structure was similar to that present in control (Fig. 8) with well-defined polymerization (Fig. 10E,e’,’’, yellow arrowheads). Viral antigen was still present and expressed by bright fluorescence in each neuronal cell (Fig. 10E,e’ green arrowhead; e’’ green arrowhead, yellow box, green arrowhead), especially in the perinuclear space of the soma (Fig. 10E,e’ yellow box; e’’ green arrowhead). The cultured primary murine neurons were not degraded after 168 h p.i. MHV-JHM.

### Effect of specific cytoskeleton inhibitors on MHV-JHM replication

**Fig. 11:**
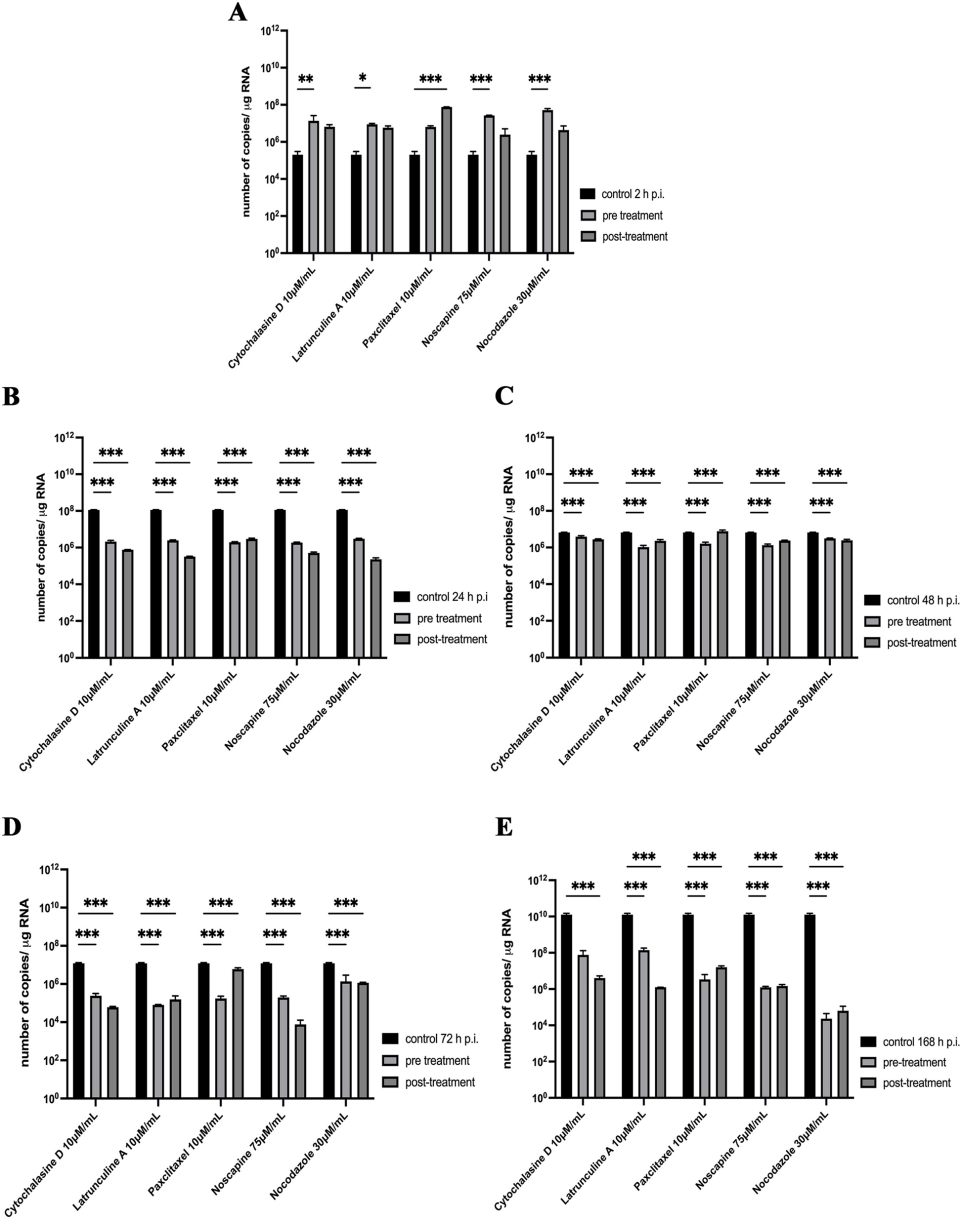
The effect of pre-treatment and post-treatment incubation with cytoskeletal inhibitors on MHV-JHM replication in primary murine neurons culture. Presented time points: 2 h (**A**), 24 h (**B**), 48 h (**C**), 72 h (**D**), 168 h (**E**). Viral RNA was quantified by RT real-time PCR. Data are presented as the mean ± standard deviation (SD) (*n* = 3). Two-way Anova tests were used to analyse statistical significance compared with the untreated control: *, significant (*P* ≤ 0.05); **, highly significant (*P* ≤ 0.01); and ***, extremely significant (*P* ≤ 0.001)

Reverse transcriptase real-time PCR was applied to detect viral RNA in neuronal cells pre-treated and post-treated with actin and microtubule inhibitors to determine their effect on virus replication. The following substances were used: cytochalasin D 10uM/mL; latrunculin A 10uM/mL; paclitaxel 10uM/mL nocodazole 30uM/mL; noscapine 75uM/mL. Both treatment methods had a significant effect on replication. Starting from 2 h p.i. there were visibly higher levels of viral RNA copies than in control untreated cells (2.02 × 10^5^) (Fig. 11A). After 24 h p.i., the viral copies decreased by at least 2 logarithms. The actin cytoskeleton inhibitors – cytochalasin D and latrunculin A had a similar effect on replication inhibition, but post-treatment method had better results. Compared to the positive control, viral RNA copies were at a level of 10^8^, whereas after applying actin depolymerization agents, latrunculin A and cytochalasin D, the values had reached level of copies 10^7^ in pre-treatment and 10^5^ in post-treatment. Cytochalasin (pre-treatment 7.47 × 10^7^; post-treatment 4.04 × 10^6^), latrunculin A (pre-treatment 1.38 × 10^8^; post-treatment 1.25 × 10^6^). Approximate values were detected for microtubule destabilization agents (Fig. 11B). In 48 h p.i. there was no spectacular effect both from actin and microtubular inhibitors. Overall positive control viral RNA copies were at a relatively high level of 10^8^ (1.13 × 10^8^) logarithm. After incubation best results were obtained for latrunculin A post-treatment (2.45 × 10^6^) (Fig. 11C). On the contrary, 72 h p.i. with extreme significance, microtubule shortening noscapine, post-treatment (7.6 × 10^3^), decreased viral replication by 4 logarithms compared to untreated control (1.2 × 10^7^). Similar results were obtained with post-treatment actin depolymerizing cytochalasin D (6.1 × 10^4^). Other pair of actin and microtubule inhibitors, latrunculin A, and paclitaxel, arrested viral replication on levels of 8.0 × 10^4^ pre-treatment, 1.6 × 10^5^, and 1.7 × 10^5^ pre-treatment, 5.9 × 10^6^ post-treatment as followed (Fig. 11D). Overall, the viral replication levels drop happened at 168 h p.i. where levels decreased by 6 logarithms after usage of post-treatment (6.3 × 10^4^) and pre-treatment (2.3 × 10^4^) incubation with nocodazole. Control untreated cells, infected with MHV-JHM (168 h p.i.), have reached value of 10^10^. Other microtubule inhibitors - noscapine and post-treatment actin depolymerization substations cytochalasin D, and latrunculin A have blocked replication by 2/3 logarithm (Fig. 11E).

## Discussion

Several works have examined and confirmed the cell’s cytoskeleton’s significant role in the virus entry into the cell and in the further stage of the replication cycle, culminating in the assembly and release of progeny virions. Much is already discovered in the context of coronaviruses, but unknown areas still leave doubts. We have delved into this topic, not least because of the increasingly reported cases of long-Covid in the CNS [43–45]. As is well known, MHV, especially neurotropic strains, e.g., JHM, A549, are well suited as models for SARS-CoV-2 [34]. Moreover, morphological, and molecular analysis of the effect of MHV-JHM infection on the cytoskeleton of primary neurons adds to the knowledge of beta-coronaviruses. Our study confirmed that MHV-JHM in 1 week infection period does not destroy neuronal cells and manipulate the cytoskeleton from entry until viral shedding. What is more important MHV-JHM uses tunnelling nanotubules as a cell-to-cell transport route avoiding immune response and direct receptor binding.

During neuroinfection, the cytoskeleton plays a very significant role. In our study, we presented a morphological and quantitative analysis verifying the level of viral replication after treating cells with substances that affect the distribution of actin filaments and microtubules. There is little information available on the utilization of the neural cell cytoskeleton by MHV-JHM. The available literature has focused more on the role of microtubules during intracellular transport than actin filaments. Microtubules have been shown to play an important role in intraneuronal transport in primary culture cells of rat hippocampal neurons and OBL-21 cells (olfactory bulb cultures of CD.1 mouse) [46]. Moreover, the team of Pasick et al. 1994 and Kalicharran and Dales, 1995 proved that there is a special interaction between Tau protein and MHV-JHM nucleocapsid protein [23, 46]. The amino acid complementarity of the two proteins overlapped at 44% similarity and 22% identity in structure. This homology probably influences such high neuropathogenicity and tropism of the virus through the interaction of MTs with MHV-JHM N protein. Some α-coronaviruses like TGEV (transmissible gastroenteritis coronavirus), HCoV-NL63 (human coronavirus NL63), and HCoV-229E (human coronavirus 229E) were proven to interact with their S and M proteins directly or indirectly with tubulin [47]. In our study, we confirmed the dominant role of microtubules during MHV-JHM infection. We already observed at 24 h p.i. moving viral particles between neurons in long axonal protuberances and neurites (Fig. 10B, green arrowheads and yellow boxes). On the other hand, at 2 h p.i. (Fig. 10A, a’’ yellow box) and 72 h p.i. (Fig. 10D, d’’ yellow and green arrowheads), MHV-JHM disrupted microtubules’ architecture, leading to their depolymerization, cell degradation, and syncytia formation (Fig. 10C, D yellow arrowheads). However, some cells were still able to transport progeny virions (Fig. 10C, D green arrowheads). Neurites did not disappear, and virions accumulated in characteristic cisternae (Fig. 10D, purple arrowheads). At 168 h p.i., the viral antigen was still present in large amounts and colocalized with the structures of the microtubule, which were intact, and the cells were not degraded (Fig. 10E). Similar results were obtained by Pasick et al. where viral antigen were present in large amounts after 48 h p.i. Virions located in a linear position were moving along axons and have formed microtubules into cisternae structures [46]. On the other hand, a study by Biswas and Sarma [48], using demyelinating strains of MHV - RSA59 and non-demyelinating RSMHV2 in the infection of Neuro2a cells, showed that viral transport took place in axonal terminals as early as 4h 15 min p.i. in the case of RSA59 and RSMHV2 in general, up to 36 h p.i. when the cells lysed. Syncytia were present as early as 9 h p.i. in Neuro2a cells upon RSA59 infection [48]. We also detected syncytium formation by MHV-JHM after 24 h p.i. as a cytopathic effect in primary murine neurons (Fig. 10B, b’’, white arrowhead), contrary to the previous finding by Bender et al. [49]. Thus, it proves that direct cell-to-cell spread in neurons may be the top way of virion spread because of its efficiency and no engagement with cell-membrane-specific receptors [50, 51]. Also, after a week of infection, we observed the renovation of the cytoskeletal structure of neurons and an increase in proliferation. Generally, infection with MHV may lead to Tau phosphorylation by glycogen synthase kinase-3β-dependent mechanism, which disrupts MT stabilizing ability causing brain damage but not neurons death [52, 53]. The results were observed by analysis of confocal microscopy images were confirmed by RT-qPCR. After pre-, and post-treating with microtubules depolymerizing agent – nocodazole, a stabilizing agent – noscapine, and paclitaxel, we have observed successful limitation in MHV-JHM replication (Fig. 11). This indicates that disrupted MTs distribution, mostly in late hours post infection (a significant number of RNA copies decreased at 72 h p.i.) had a highly statistical effect on the virus’s ability to replicate in primary murine neurons. The best results were obtained by noscapine 72 h p.i. post-infection treatment by three logarithms drops and nocodazole pre-treatment by six logarithms drop (Fig. 11). In other viruses, the replication of sindbis virus, vesicular stomatitis virus, and human herpes virus type I, was quantified by the titre (plaque forming units/ml; pfu/ml) produced in cells treated with three anti-microtubule drugs (colchicine, noscapine, or paclitaxel) and none of these drugs affected the replication [54]. In the case of MHV-A59 and MHV-2 treatment with colchicine, vinblastine did not affect fusogenic properties and thus replication in 36 h p.i. in Neuro2a cells and fibroblasts [48, 55].

To our knowledge, no publications considered changes in actin filament during MHV-JHM infection in neurons. We have observed dynamic rearrangement of F-actin which led to facilitated MHV-JHM entry into the soma (Fig. 5). It is well known that microfilaments participate in virion surfing after the virus binds to a target cell [54]. In our study, as early as 2 h post infection, the filament condensation in the form of submembranous rings were seen (Fig. 5A,a’’ green arrowhead; Fig. 6). These structures, which can be sites of viral entry, were also present after IPEC-J2 cells infection with PEDV (Porcine epidemic diarrhoea virus) and TGEV (Transmissible gastroenteritis coronavirus) [56]. The first changes appeared 2 h p.i., where excessive condensation of actin in the submembranous region and its thinning in the zone close to the cell nucleus (Fig. 9A, green arrows) were noted. We also captured the penetrating/moving viral antigens in the long filopodial protuberance (Fig. 9A, white arrowhead) and the accumulation of virion antigen in the perinuclear area. The changes worsened at 24 h p.i. when productive replication occurred. Neurons formed numerous intercellular connections in the form of longitudinal actin filaments, probably forming tunnelling nanotubes for transporting viral particles. The use of TNTs and their important involvement in intercellular transport during SARS-CoV-2 infection has recently been discovered. The enhanced ability to form TNTs and the movement of viral antigen within the bridges were investigated on a coculture model of African green monkey kidney Vero E6 cell and human neuroblastoma (SH-SY5Y) cells [57]. In our study conducted on primary murine neurons, numerous TNT structures were seen and the movement of MHV-JHM antigen within TNTs throughout the infection period were noted. Interestingly, the largest number of this actin structures appeared after 168 h p.i. which may be an important sign especially in the treatment or research for the pathology of long-COVID and the changes that coronaviruses cause in the central nervous system.

Peripheral stress fibres in the form of highly condensed fibres were visible, and a large amount of viral antigen localized in the perinuclear region (Fig. 9B, white arrowheads) with a much-diluted structure was still clearly visible. Like in microtubule structure, proper actin filaments dynamics were restored after 168 h p.i. (Fig. 9E). During later hours post infection, characteristic juxtanuclear rings were observed. Again, in PEDV and TGEV infected IPEC-J2 cells, juxtanuclear rings support viral genome replication and protein synthesis [56]. Actin filaments also promote viral egress by thickening and restoring stress fibres structures, as we noted. Such phenomenon occurred during IBV (Infectious Bronchitis Virus) and SARS-CoV infection and proved essential during virus budding and assembly of viral particles [31, 58, 59]. To confirm the role of the actin cytoskeleton in MHV-JHM infection, we have used cytochalasin D and Latrunculin A treatment (Fig. 11). These two actin polymerization inhibitors did not influence the entry of the virus, thus the replication ability of MHV-JHM after 2 h p.i., but in later stages of replication, the inhibitory effect was visible by three logarithms decreased after 168 h p.i. In a study by Yeung 2021, BafA1 and cytochalasin D, which impair endosomal acidification and endosomal-lysosomal system, respectively, were proven to inhibit the infection of the SARS-CoV-2 virus [60].

In conclusion, here, we showed that neuronal actin cytoskeleton is likely used during MHV-JHM infection, which we confirmed by morphological analysis. Its role is not essential in the process of viral penetration but crucial in cell-to-cell transport of progeny virions. This type of viral transport permits infected cells to hide from immune system response and allows more effective viral spreading. Our studies show that the primary role in the intracellular transport of MHV-JHM virions to the site of replication and then between cells is played by microtubules. This finding was confirmed after using noscapine and nocodazole inhibitors, which effectively reduced MHV-JHM replication in neurons.

## Data Availability

The datasets used and/or analysed during the current study are included in this publication.
